# Quantifying the impact of heat on human physical work capacity; part II: the observed interaction of air velocity with temperature, humidity, sweat rate, and clothing is not captured by most heat stress indices

**DOI:** 10.1007/s00484-021-02212-y

**Published:** 2021-11-06

**Authors:** Josh Foster, James W. Smallcombe, Simon Hodder, Ollie Jay, Andreas D. Flouris, George Havenith

**Affiliations:** 1 Environmental Ergonomics Research Centre, Loughborough University, Loughborough, LE11 3TU UK; 2 Thermal Ergonomics Laboratory, Faculty of Medicine and Health, The University of Sydney, Sydney, NSW Australia; 3 FAME Laboratory, University of Thessaly, Trikala, Greece

**Keywords:** Heat, Productivity, Fans, Wind, Convection, Sweating

## Abstract

Increasing air movement can alleviate or exacerbate occupational heat strain, but the impact is not well defined across a wide range of hot environments, with different clothing levels. Therefore, we combined a large empirical study with a physical model of human heat transfer to determine the climates where increased air movement (with electric fans) provides effective body cooling. The model allowed us to generate practical advice using a high-resolution matrix of temperature and humidity. The empirical study involved a total of 300 1-h work trials in a variety of environments (35, 40, 45, and 50 °C, with 20 up to 80% relative humidity) with and without simulated wind (3.5 vs 0.2 m∙s^−1^), and wearing either minimal clothing or a full body work coverall. Our data provides compelling evidence that the impact of fans is strongly determined by air temperature and humidity. When air temperature is ≥ 35 °C, fans are ineffective and potentially harmful when relative humidity is below 50%. Our simulated data also show the climates where high wind/fans are beneficial or harmful, considering heat acclimation, age, and wind speed. Using unified weather indices, the impact of air movement is well captured by the universal thermal climate index, but not by wet-bulb globe temperature and aspirated wet-bulb temperature. Overall, the data from this study can inform new guidance for major public and occupational health agencies, potentially maintaining health and productivity in a warming climate.

**Supplementary Information:**

The online version contains supplementary material available at 10.1007/s00484-021-02212-y.

## Introduction

The association between climate change and hot weather patterns is now well established (Mazdiyasni et al., 2019; Zhang et al., 2020). Increased human exposure to more prolonged, frequent and intense heat negatively impacts productivity in physically demanding occupations (Ioannou et al., 2017; Mairiaux and Malchaire, 1985; Messeri et al., 2019; Miller et al., 2011; Morrison et al., 1969; Sahu et al., 2013; Wyndham, 1973). Consequently, labour loss constitutes a major factor towards the economic damage associated with global warming (Hsiang et al., 2017).

Our group recently adopted a laboratory based protocol in which occupational physical work capacity (PWC) was evaluated in a wide range of environmental conditions (Foster et al., 2021a). We defined PWC as “the maximum physical work output that can be reasonably expected from an individual performing moderate to heavy work over an entire shift” (Foster et al., 2021a). Our group developed empirical models which precisely estimate PWC from a wide range of air temperature and relative humidity combinations (Foster et al., 2021a), including correction factors which account for individual characteristics (fitness, age, and sex) (Foster et al., 2021c) and solar irradiance (Foster et al., 2021b). Another major task was to evaluate the impact of increasing wind speed on PWC, which can likely be beneficial or detrimental depending on the specific air temperature (*T*_a_) and humidity combination. The present paper reports on these findings.

Increased air movement, derived from natural wind or artificially induced with electric fans, can primarily impact convective and evaporative heat transfer pathways. In an occupational work context, convection refers to heat picked up from the skin by surrounding air, where the rate is proportional to the wind speed (Havenith et al., 1990b; Holmér et al., 1999) and the temperature gradient between skin and environment. Human skin temperature (*T*_skin_) is typically not lower than ~ 33 °C in warm environments, so when *T*_a_ ≤ 30 °C, increasing the movement of cooler air over warm skin picks up heat and provides a cooling effect. Adoption of fans ≤ 30 °C *T*_a_ has been shown to increase physical work capacity (Jay et al., 2019; Wyndham, 1969) and improve thermal comfort in offices (Atthajariyakul and Lertsatittanakorn, 2008; Schiavon et al., 2017), decreasing the requirement for energy-demanding air conditioning units. Fans should therefore always be recommended for reducing occupational heat strain when *T*_a_ ≤ 30 °C (regardless of humidity), with diminishing benefits from convective heat loss as *T*_a_ approaches *T*_skin_.

When *T*_a_ reaches and exceeds *T*_skin_ (~ 35 °C), convective heat loss is reversed into heat gain, and increased air movement with fans can force additional heat back into the body. This physical principle likely underpins advice from major health agencies such as the (World Health Organisation 2020); (Centre for Disease Control and Prevention, 2020), and (Public Health England, 2018), who, at the time of writing this paper, advise against using fans when *T*_a_ ≥ 35 °C. However, recent empirical evidence in resting humans would suggest these guidelines are too conservative, because they do not account for how electric fans impact *evaporative* heat transfer (Jay et al., 2015; Ravanelli et al., 2015, 2017). One gram of evaporated sweat has the potential to liberate up to ~ 2430 J of heat energy from the body, and like the convective pathway, the rate of evaporative heat loss is directly proportional to the speed of air movement over the skin (Havenith et al., 1990a, 1999). Increasing wind speed can improve the rate of heat loss by impacting sweat evaporative efficiency (Candas et al., 1979b), increasing the proportion of sweat that evaporates (providing cooling), compared with sweat that the drips off the body (providing no cooling) (Candas et al., 1979b). Since sweating efficiency is already high in arid climates with low water vapour pressure (Candas et al., 1979a), but lower in humid climates, high wind appears to be most beneficial in the latter condition (Morris et al., 2019). Indeed, early predictions using biophysical calculations suggests that high wind/fans would always have a net cooling effect if the speed is ≥ 2.5 m∙s^−1^, regardless of the temperature and humidity combination (at least up to 45 °C *T*_a_) (Gagge et al., 1937). However, the empirical data thus far suggest that wind/fans are beneficial in warm humid climates, but may be detrimental in hot dry ones (discussed below).

Empirical studies demonstrate potential cooling effects of fans up to ~ 42 °C *T*_a_ at rest (Morris et al., 2019; Ravanelli et al., 2015, 2017), with a strong dependency on humidity. For example, electric fans provided a net cooling effect in a hot humid environment (40 °C *T*_a_, 50% relative humidity) but were detrimental in very hot and arid conditions (47 °C *T*_a_, 10% relative humidity) (Morris et al., 2019). These findings disagree with early simulated data discussed above (Gagge et al., 1937). Additional studies in resting participants have found a net cooling effect of fans at 36 and 42 °C *T*_a_ (Ravanelli et al., 2015, 2017), with upper relative humidity limits of 80 and 50%, respectively (Ravanelli et al., 2015, 2017). These limits are further extended with the addition of water spraying on the skin (Hospers et al., 2020). These studies indicate that (i) fans can provide a net cooling effect above current recommended limits and (ii) humidity must be considered when providing recommendations for public policy. Nevertheless, this prior work investigating the impact of fans ≥ 35 °C *T*_a_ has only involved resting participants and only assessed the effect of fans in a limited number of environments.

Combining a large number of empirical observations with a computational/biophysical model of human heat transfer should allow the generation of practical advice, especially for occupational and public health sectors. If the model is shown to be valid (i.e. follows the response of the empirical trials), it may be used to predict the impact of high wind/fans on PWC outside of the specific conditions tested in this experiment. Moreover, its input parameters (i.e. maximum sweat rate and wind speed) can be modified to provide more appropriate guidance based on the population of interest (i.e. old vs young, heat acclimatized vs non-acclimatized) or wind speed differences. A second issue of interest is the ability of unified weather indices to predict any change in human performance in high wind environments. Among the most popular indices are the universal thermal climate index (UTCI), the wet-bulb globe temperature (WBGT), and psychrometric/aspirated wet-bulb temperature (*T*_wb_) (Havenith and Fiala, 2015; Stull, 2011). The UTCI is based on a complex thermophysiological model (Fiala et al., 2012) and, as such, is expected to account for any changes in wind speed and its interaction with the thermoregulatory system, i.e. it may predict a net beneficial or detrimental effect of high wind depending on the ambient environment. Conversely, the WBGT value can by definition only *decline* in high wind conditions, due to the increased water evaporation from the wick and potential cooling of the black globe, such that it may not account for the *environment dependent* impact of wind shown previously (Morris et al., 2019). This is highly relevant since WBGT is the most widely used heat stress metric in industry (Havenith and Fiala, 2015). The aspirated *T*_wb_ is calculated based on air temperature and relative humidity alone (Stull, 2011) and, as such, does not change/account for variations in air movement. It therefore is unlikely to be an appropriate heat stress metric for predicting human PWC in high wind conditions, despite its widespread use in predicted human heat strain on a global scale (Raymond et al., 2020).

The present study builds on this prior work to improve our knowledge on the impact of air movement on heat strain at moderate to heavy workloads, with the goal to inform new guidelines for industry. Using empirical trials and a biophysical model of human heat transfer, we investigate the impact of wind/fans on heat storage and physical work capacity across wide variations in *T*_a_ and relative humidity, covering almost all relevant climate types for those working in hot environments.

## Methodology

We undertook a comprehensive empirical study, collecting data from 23 participants in 300 trials (24 cool reference trials, 138 hot still air trials, 138 hot fan trials) to determine how fans (3.5 m·s^−1^ air speed) impact physical work capacity (PWC) under sixteen *T*_a_ and relative humidity combinations (35–50 °C *T*_a_, 20–80% relative humidity), during a 1-h paced work simulation in a climatic chamber. The hot trials were separated into 154 trials with low clothing coverage (shorts and trainers, ~ 0.28 Clo) and 121 with higher clothing coverage (full body coverall, t shirt, shorts, trainers, ~ 0.86 Clo). We further expanded the scope of the advice by generating a biophysical model, using a human heat balance approach. The model calculates the impact of fans on heat storage (*S*, in W·m^−2^) for any given combination of *T*_a_ and relative humidity. Ethical approval was provided by the Loughborough University Research Ethics Committee.

### Empirical study

#### A fixed heart rate approach simulates self-pacing during physical work

The physical work simulation was based on the observations that self-paced physical work is governed largely by a stable working heart rate, regardless of the heat stress severity (Bates and Schneider, 2008; Brake and Bates, 2001; Kalkowsky and Kampmann, 2006; Mairiaux and Malchaire, 1985; Miller et al., 2011). We aimed to simulate this pacing strategy by fixing the working heart rate (and thus, cardiovascular strain) across all tested conditions of *T*_a_ and relative humidity. This fixed heart rate protocol has been used previously to determine the effect of fans on light work output at 30 °C *T*_a_ (Jay et al., 2019), and for modelling the impact of heat stress and physical work capacity (Foster et al., 2021a, b, c). During physical work, skin blood flow requirements increase as a function of heat stress severity (Rowell, 1974), resulting in a progressively larger proportion of cardiac output being directed to the skin (to support heat loss) compared with muscle (to support physical work). Consequently, for a fixed heart rate, less physical work can be achieved in hot climates when compared with cool climates, as observed in the field (Kalkowsky and Kampmann, 2006; Miller et al., 2011). Based on the assumption that the maximum average work level and cardiovascular load that might be reasonably expected from a worker falls between the “moderate” and “heavy” domains (Brake and Bates, 2001) as classified by the WHO (125 to 130 beat·min^−1^ for a 25–30-year-old) (Andersen, 1978), the energy generated above resting levels during a work bout in this intensity domain can be considered the maximal achievable capacity for prolonged physical work. Measuring work output (total kilojoules, kJ) in a cool reference environment (15 °C, 50% relative humidity, *Cool*_kJ_) and then under various heat stress conditions (*Hot*_*kJ*_) allowed us to calculate percentage changes to PWC due to heat and the subsequent impact of electric fans in a *within participants* design:

1 $${\varvec{P}}{\varvec{W}}{\varvec{C}}= \left(\frac{Ho{t}_{\mathrm{kJ}}}{Coo{l}_{\mathrm{kJ}}}\right)\times 100$$

where *Cool*_kJ_ is the total energy generated (kJ) above resting metabolism in the cool reference condition (15 °C, 50% relative humidity) and *Hot*_kJ_ is the total energy generated above resting metabolism in each heat stress trial. All participants arrived in a euhydrated state. They were not permitted to drink water during the 1-h trial, and a progressive dehydration throughout the trial may also contribute to increased heart rate (and less physical work) during heat exposures (Gonzalez-Alonso et al., 1995). However, sweat rate was largely similar in all trials *within subjects* due to adjustments in required evaporation that accompany any changes in metabolic work rate.

#### Participants

The characteristics of those in the empirical study are detailed below: A detailed description of how these variables were derived is available in the section “Preliminary trial (Visit 1)” (Table 1) .

**Table 1:** Participant characteristics in the empirical study. Data are presented as mean ± standard deviation. The range is presented below in parentheses

Age	Height	Body fat	BMI	BSA	*V̇*O_2max_	*V̇*O_2max_	Mass
Years	cm	%	kg·m^−2^	m^−2^	L·min^−1^	mL·kg^−1^·min^−1^	kg
***Semi-nude* (*n* = 15)**							
25 ± 3	178 ± 5	19 ± 5	24 ± 2	1.9 ± 0.1	3.7 ± 0.7	49 ± 8	75 ± 10
(19 – 28)	(172–190)	(10–26)	(20–28)	(1.7–2.3)	(2.8–5.1)	(40–65)	(59–101)
***Coverall* (n = 13)**							
23 ± 2	177 ± 5	14 ± 5	23 ± 3	1.9 ± 0.1	3.9 ± 0.7	54 ± 8	73 ± 9
(19–28)	(172–186)	(8–26)	(21–30)	(1.7–2.1)	(3.0–5.3)	(41–67)	(63–95)

*V̇*O_2max,_ maximal oxygen consumption; *BMI* body mass index; *BSA* body surface area; *L* litres

#### Experimental controls

Participants completed trials at the same time of day to reduce any possible effect of diurnal variation on thermoregulatory variables (Waterhouse et al., 2004). Participants arrived hydrated for each trial, did not consume caffeine on the day of each trial, and did not perform rigorous exercise or consume alcohol at least 24 h prior to each session.

#### Preliminary trial

Body mass was quantified to the nearest gram using a digital scale (Metter Toledo kcc150, Metter Toledo, Leicester, UK), with height measured to the nearest 0.5 cm with a stadiometer (Holtain, Crosswell, UK). Body composition was measured with bioelectrical impedance (Tanita MC-780MA, TANITA Corporation, Tokyo, Japan).

A treadmill-based submaximal exercise test was performed on a treadmill in a regulated environment of 18 °C, 40% RH (Mercury Medical, h/p/cosmos sports & medical Gmbh, Germany). The test involved up to 6 stages lasting 3 min each. The test used a fixed speed of 4.5 km·h^−1^, while the gradient increased by 5% after each stage. Expired air (*V̇*O_2_) and heart rate were continuously measured indirect calorimetry (Quark CPET, COSMED, Albano Laziale, Rome) and telemetry (Polar PE4000, Polar Electro, Kempele, Finland), respectively. The test was terminated when steady state heart rate reached 85% of age predicted maximum. The *V̇*O_2_ and heart rate data collected during the submaximal treadmill test were extrapolated to estimate *V̇*O_2max_ (ACSM, 2013).

#### Experimental trials

Rectal temperature was measured continuously in this study as a safety measure. Participants were removed from the climate chamber and the trial terminated if core temperature reached 39 °C. This occurred on 2 occasions out of 300 trials (0.67% of trials). Upon arrival, participants provided a urine sample and inserted a rectal thermometer (VIAMED, Yorkshire, UK) to a depth of 10 cm. If the urine sample showed a specific gravity of > 1.020, participants consumed 0.5 l of water and provided a second urine sample after 20–30 min (Armstrong et al., 1994). The skin temperature response to variations in air temperature and relative humidity were used for human heat balance modelling (see supplementary Eq. 3). Skin temperature (*T*_skin_) was measured with unidirectional skin thermistors (Grant Instruments, EUS-UU-VL-3.0, Dorset, UK). Skin thermistors were taped on the belly of the *pectoralis major* (*T*_chest_), *triceps* (*T*_arm_), *rectus femoris* (*T*_thigh_), and *gastrocnemius* (*T*_calf_). The mean weighted *T*_skin_ was calculated based on Ramanathan (1964). The value for *T*_skin_ was reported as the mean score throughout each trial.

2 $${\varvec{S}}{\varvec{k}}{\varvec{i}}{\varvec{n}}\boldsymbol{ }{\varvec{T}}{\varvec{e}}{\varvec{m}}{\varvec{p}}{\varvec{e}}{\varvec{r}}{\varvec{a}}{\varvec{t}}{\varvec{u}}{\varvec{r}}{\varvec{e}}=0.3\left({T}_{\mathrm{chest}}+{T}_{\mathrm{arm}}\right)+0.2\left({T}_{\mathrm{thigh}}+{T}_{\mathrm{calf}}\right) \left[^\circ C\right]$$

The empirical whole body sweat loss (WBSL) response was used in the biophysical model (see supplementary Eq. 15).WBSL was calculated from the change in nude body mass pre and post heat exposure:

3 $${\varvec{W}}{\varvec{B}}{\varvec{S}}{\varvec{L}}= Pre \; body \; mas-Post \; body \; mass \left[\mathrm{g}\right]$$

Participants entered the environmental chamber wearing light clothing (0.26 Clo) or work clothing (0.86 Clo). The heat transfer properties of each ensemble are described in the supplementary material and in our recently published work (Foster et al., 2021a).

Various data acquisition systems were used to log skin and core temperature (Grant Squirrel SQ2020, Grant Instruments Ltd, Corby, UK), Wet-bulb globe temperature (WBGT) (Quest temp model 34), air temperature, relative humidity, and air velocity (Testo Ltd, model 435–2 Alton, Hampshire, UK) at 1-min intervals.

#### Physical work simulation

The treadmill (h/p Cosmos Mercury) was programmed to maintain a constant heart rate of 130 b·min^−1^ by controlling speed and grade. To avoid jogging exercise, once speed reached a predefined limit of 6 km·h^−1^, elevation was adjusted based on the deviation between measured heart rate and set point heart rate (130 b·min^−1^). While the maximum test duration was 1 h, the trial did not continue if the treadmill speed fell to zero, i.e. the participant could not maintain a heart rate < 130 b·min^−1^ while standing. The work output achieved up until the point of cessation was used as the final cumulative kilojoule (kJ) output (shown in Eq. 4). Table S1 in the supplementary material shows the average trial durations for each environment.

#### Calculation of percentage physical work capacity (PWC)

Physical work capacity (%) was calculated based on the total energy expenditure (EE) above resting in each trial, relative to that achieved in a cool reference condition (see Eq. 1). The rate of work EE in kJ·min^−1^ during each minute of work was calculated as below in Eq. 4, with the cumulative total used as final work EE (in kJ) (Ludlow and Weyand, 2017):

4 $$Work EE={\sum }_{t=1}^{60}\left[0.32\cdot G(t)+3.28+\left(1+0.19\cdot G(t)\right)\cdot \left(2.66\cdot {v(t)}^{2}\right)\right]\cdot \left[19.61+\frac{RQ(t)-0.707}{0.293}\cdot 1.51\right] [kJ]$$

where *G(t)* denotes the treadmill percentage grade at time t, *v(t)* is walking speed in m·s^−1^, and *RQ(t)* is respiratory quotient and was assumed to be 0.85 (Cramer and Jay, 2019). ∑ denotes that the output of the equation is summed every 1 min (*t* = 1) until the cessation of each trial. Part 1 (first square bracket) of the equation (Ludlow and Weyand, 2017) quantifies the net volume of oxygen consumed (*V̇*O_2-net_, in mL·kg body mass^−1^·min^−1^) to fuel exercise, i.e. not including resting *V̇*O_2_. Part 2 of the equation (second square bracket) converts the former into kJ.min^−1^. The cumulative EE for each trial was used to calculate total EE (in kJ) in each individual trial. The validity of the prediction equation for EE was confirmed in our previous work (Foster et al., 2021a).

#### Environment selection

The purpose of the study was to evaluate the impact of high wind/fans on PWC at *T*_a_ = 35 °C and above. Below this air temperature (i.e. at 30 °C), fans have already been shown to improve work output at a fixed heart rate (Jay et al., 2019). Due to the biophysics of human heat transfer described in the introduction, increasing the wind speed with fans will always provide a net cooling effect when *T*_a_ ≤ 30 °C. The reference condition in which net-kJ (work) output was compared against was 15 °C, 50% relative humidity, as used previously (Foster et al., 2021a). Performance in all hot trials was expressed as a percentage of that achieved in the cool reference condition.

For practicality, we investigated the impact of fans on PWC in 5 °C *T*_a_ increments (i.e. 35, 40, 45, and 50 °C). For each level of *T*_a_, we then verified the threshold relative humidity (lower and upper) by which fans have a beneficial or negative impact. At *T*_a_ = 50 °C, investigating the impact of fans above 40% relative humidity was not required, since the severity of the condition would render PWC already minimal regardless of whether fans are switched on or off. Such extreme conditions have been seldom reported to occur naturally. For this reason, the recommendations shown in the matrices (Figs. 4 and 6) are capped at 6 kPa ambient vapour pressure.

### Calculation of heat stress indices

#### Wet-bulb globe temperature (WBGT)

During the still air trials, WBGT was measured empirically using a WBGT monitor (Quest temp model 34). The value used for subsequent analysis was the average over the course of each work bout.

For the wind/fan trials, live WBGT measurement during the exercise was not practical since the WBGT meter needed to be placed in the same location as the exercising human participant. Hence, steady-state WBGT was measured on a separate occasion while exposed to the same conditions.

#### **Aspirated (psychrometric) wet bulb temperature (T**_**wb**_**)**

Psychrometric wet-bulb temperature was calculated as (Stull, 2011):

5 $$\begin{array}{cc}{T}_{wb}={T}\cdot{atan}\left[0.151977{\left(Rh+8.313659\right)}^{1/2}\right]+\mathrm{atan}\left(T+Rh\right)-\mathrm{atan}\left(RH-1.676331\right)+0.00391838{\left(RH\right)}^{3/2}\mathrm{atan}\left(0.023101RH\right)-4.686035& \left[^\circ C\right]\end{array}$$

where *T* is air temperature in degrees Celsius and RH is relative humidity % (0–100).

#### Universal thermal climate index (UTCI)

The UTCI was determined using an excel calculator (www.climatechip.org/excel-wbgt-calculator), using the regression polynomial provided by the operational procedure of UTCI (Bröde et al., 2012).

### Human heat balance modelling

A human biophysical model was developed to produce a high-resolution matrix which can state, based on combinations of *T*_a_ and RH, if high wind/fan use is likely to have a beneficial, detrimental, or negligible impact on human heat storage, and therefore PWC. Following validation from the empirical data, the model can also be used to make first projections about how these zones change based on variations in wind speed and sweating rate (albeit without supporting empirical data from this study, see Fig. 6). The model was used to compare the changes in the rate of heat storage (Δ*S*) of the worker’s body for each air temperature (30–50 °C) and relative humidity (5–100% with an absolute humidity limit of 6 kPa) combination, with and without fans (i.e. 0.2 vs 3.5 m·s^−1^ air velocity). The model simulation was run based on a worker with low clothing coverage (0.28 Clo). The model is presented in the supplementary material/appendix. The model follows the heat balance calculations as described in previous works (Havenith and Fiala, 2015; ISO7933, 2004; Lotens and Havenith, 1991; Malchaire et al., 2001).

Developing a heat budget model (Cramer and Jay, 2019) based on the empirical observations was attractive but not practical in this study, due to non-steady state levels of heat production (to ensure a stable heart rate) and subsequently, unclear estimates of sweating efficiency changes over time. Although the model used in our study requires an input for metabolic rate, this does not change the *difference* in heat storage between fan off vs fan on, which was the primary outcome variable used to develop advice in Fig. 4. Instead, the primary feature of our model is the use of an inputted sweat rate, which was estimated based on the empirical data (see Eq. 15 in supplementary file) but largely similar in all environments (~ 1 L per hour). While keeping a fixed sweat rate, variations in wind speed allow for an adjustment in convective and evaporative heat transfer parameters, which ultimately impact heat storage (*S*).

The model allows calculation of the rate of heat storage (*S*) between fan off (0.2 m·s^−1^) and fan on (3.5 m·s^−1^) conditions. For fan off and fan on conditions, *S* was compared for all relevant combinations of air temperature (30–50 °C) and relative humidity (5–100%), i.e. 328 combinations. The difference in *S* was calculated as:

6 $$\mathrm{Fan \; effect }(\Delta S)= {S}_{fan-on}-{S}_{fan-off}\left[W\bullet {m}^{-2}\right]$$

where *S*_*fan-off*_ is the rate of heat storage with fans switched off (i.e. in still air) and *S*_*fan-on*_ is the rate of heat storage with fans switched on. The model was subsequently validated against our empirical dataset, i.e. the change in heat storage elicited by fans plotted against the measured change in PWC% by using fans (see Fig. 3).

## Results

### Effect of fans on PWC

**Fig. 1:**
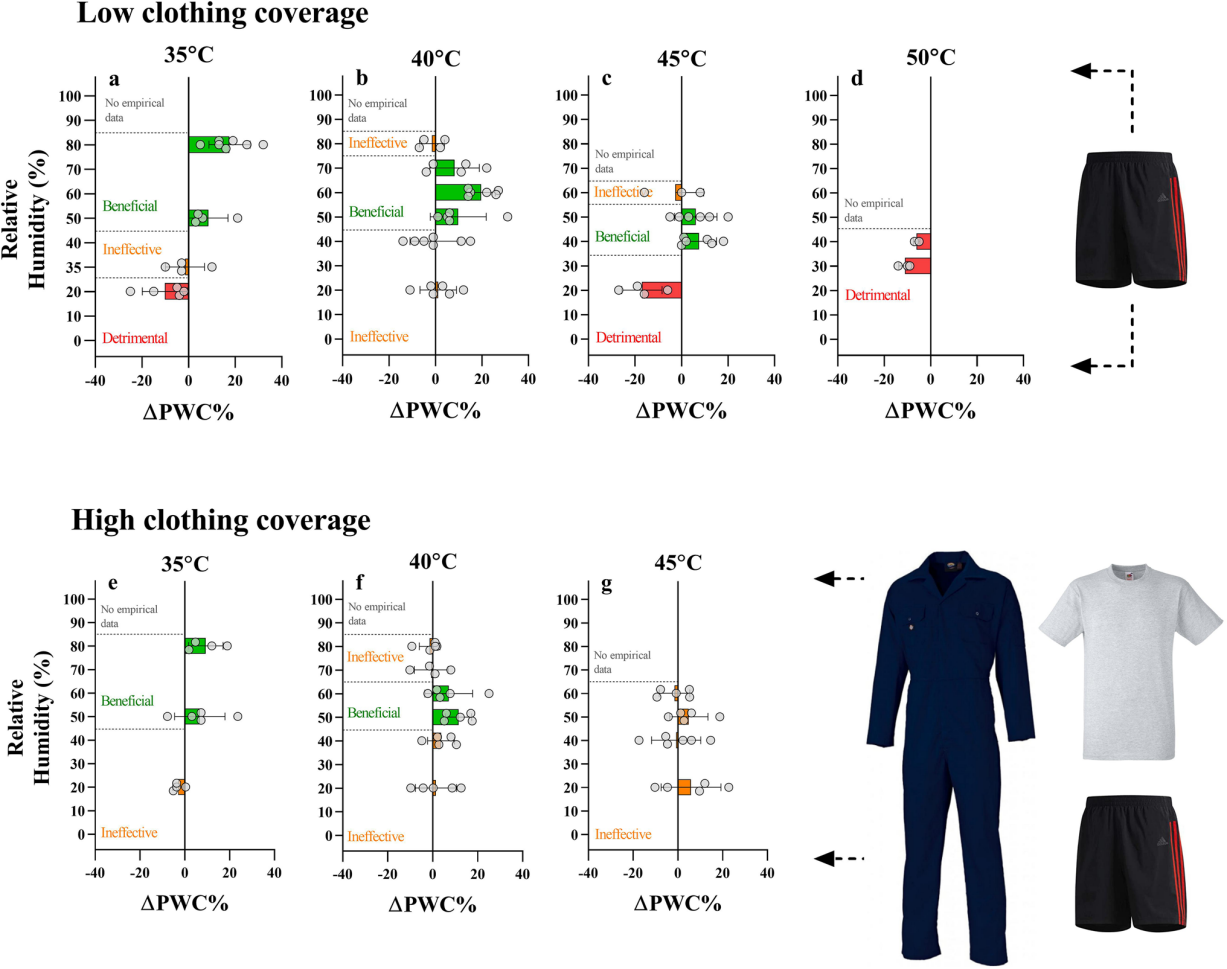
The impact of electric fans on the change in physical work capacity (ΔPWC%) with minimal clothing or work clothing in relation to temperature and relative humidity. In minimal clothing (A), depending on humidity, the extra sweat evaporation with fans was effective up to *T*_a_ = 45 °C, 10 °C higher than current guidance. A similar pattern was evident with work clothing, but fans were only beneficial up to *T*_a_ = 40 °C. “Beneficial” was defined as a mean **Δ**PWC of >  + 5% PWC, “detrimental” was defined as a mean **Δ**PWC of > -5% PWC, and “ineffective” was defined as a **Δ**PWC between − 5 and + 5%. Grey dots indicate a fan vs no fan comparison for an individual subject. Bars indicate the average response and error bars denote standard deviation

Figure 1 (a–d) shows the experimental results and demonstrates the impact of fans on PWC while working with low clothing coverage, i.e. only shorts and trainers. For a given *T*_a_ ≥ 35 °C, the effectiveness of fans on PWC is clearly dictated by ambient humidity. Improvements in PWC of ~ 20% occurred at *T*_a_ = 35 °C and 40 °C at 80 and 60% relative humidity, respectively (~ 4.5 kPa ambient water vapour pressure, *P*_a_). The ranges of relative humidity in which fans are likely effective are 45–85% (*T*_a_ = 35 °C), 45–75% (*T*_a_ = 40 °C), and 35–55% (*T*_a_ = 45 °C). Below these humidity ranges (and *T*_a_ =  > 45 °C), fans were typically detrimental, decreasing PWC. At *T*_a_ = 45 °C and 20% relative humidity, using fans decreased PWC by 17%, and fans always decreased PWC at *T*_a_ = 50 °C regardless of humidity (only relative humidity conditions that were still positive at 45 °C were tested at 50 °C). Figure 1 (e–g) reveals a similar pattern when high clothing coverage in the form of work coveralls (35% cotton 65% polyester, insulation ~ 0.86 Clo), but with benefits occurring at a narrower range (*T*_a_ = 35–40 °C) and within a range of relative humidity between 45 and 65% at *T*_a_ = 40 °C and between 45 and 85% at *T*_a_ = 35 °C.

**Fig. 2:**
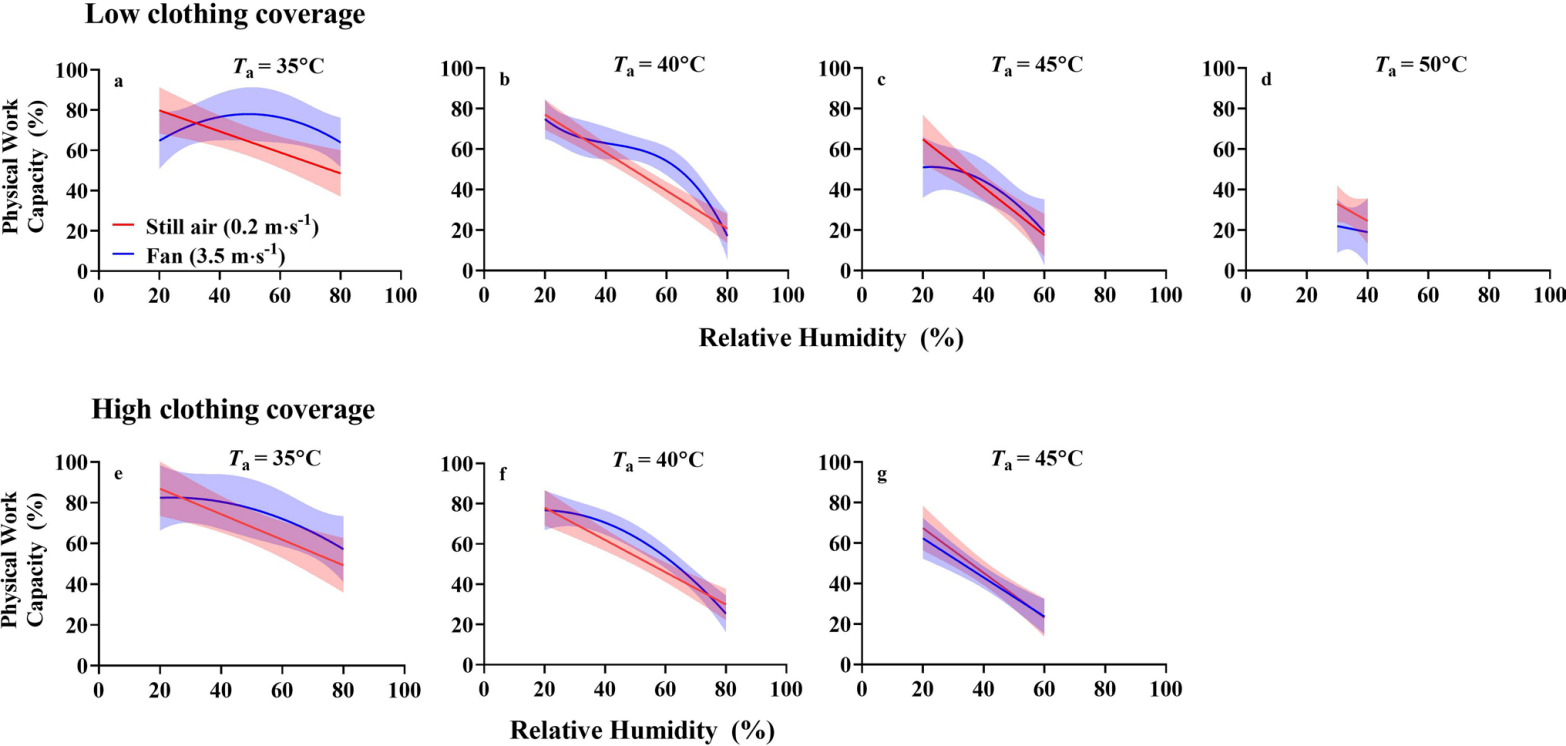
Effect of humidity on total physical work capacity (PWC) with and without electric fan use for semi-nude (**a**–**d**) and coverall (**e**–**g**) conditions. The relation between absolute PWC and relative humidity for a given air temperature (*T*_a_) is dependent on the wind speed. The red models demonstrate that PWC decreases as a linear function of relative humidity in still air conditions. The blue models demonstrate a curvilinear relation if data is plotted for higher wind speeds. Nonlinear data were modelled by polynomial regression. Electric fans generally preserve PWC when relative humidity > 50%. Shaded areas indicate 95% confidence interval bands

While Fig. 1 shows the change in physical work capacity (ΔPWC) elicited by wind/fans, Fig. 2 shows how fans modulate the absolute PWC responses to various *T*_a_ and relative humidity combinations, with and without work clothing. For any given *T*_a_ in still air, PWC decrease linearly as relative humidity increases. However, data from the fan conditions indicate a mostly nonlinear PWC response, in which fans can preserve or degrade PWC in a humidity dependent manner.

### Biophysical model validation

**Fig. 3:**
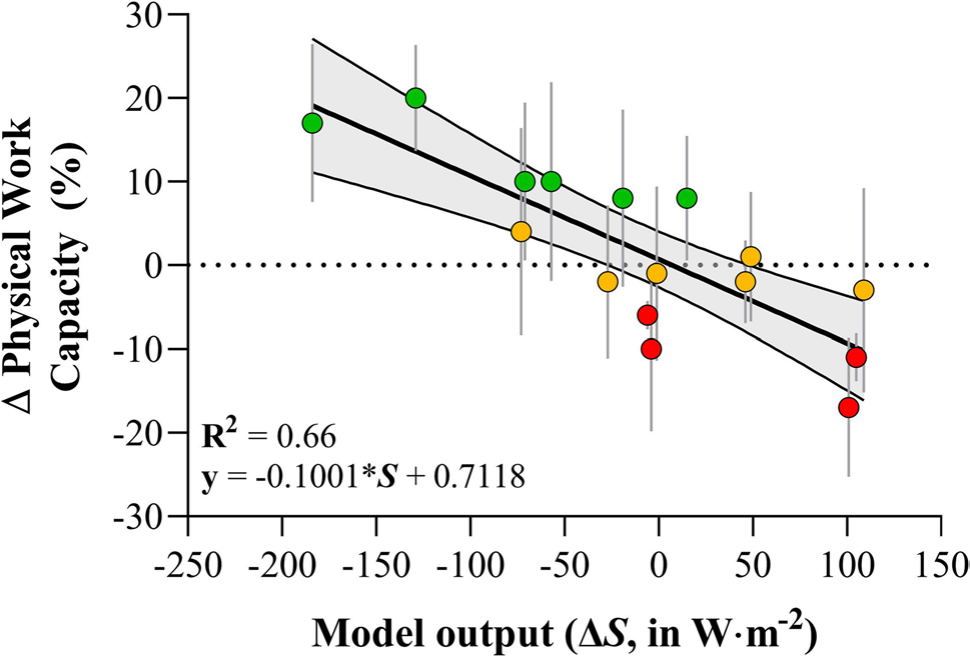
Relationship between the change in physical work capacity (ΔPWC, empirical data) and the change in heat storage rate (ΔS, biophysical model) elicited by fans. Each dot represents the group level ΔPWC, and the error bars show the standard deviation. Green dots indicate a ΔPWC of > 5%, orange symbols indicate a ΔPWC − 5 to 5%, red dots indicate a ΔPWC <  − 5%

**Fig. 4:**
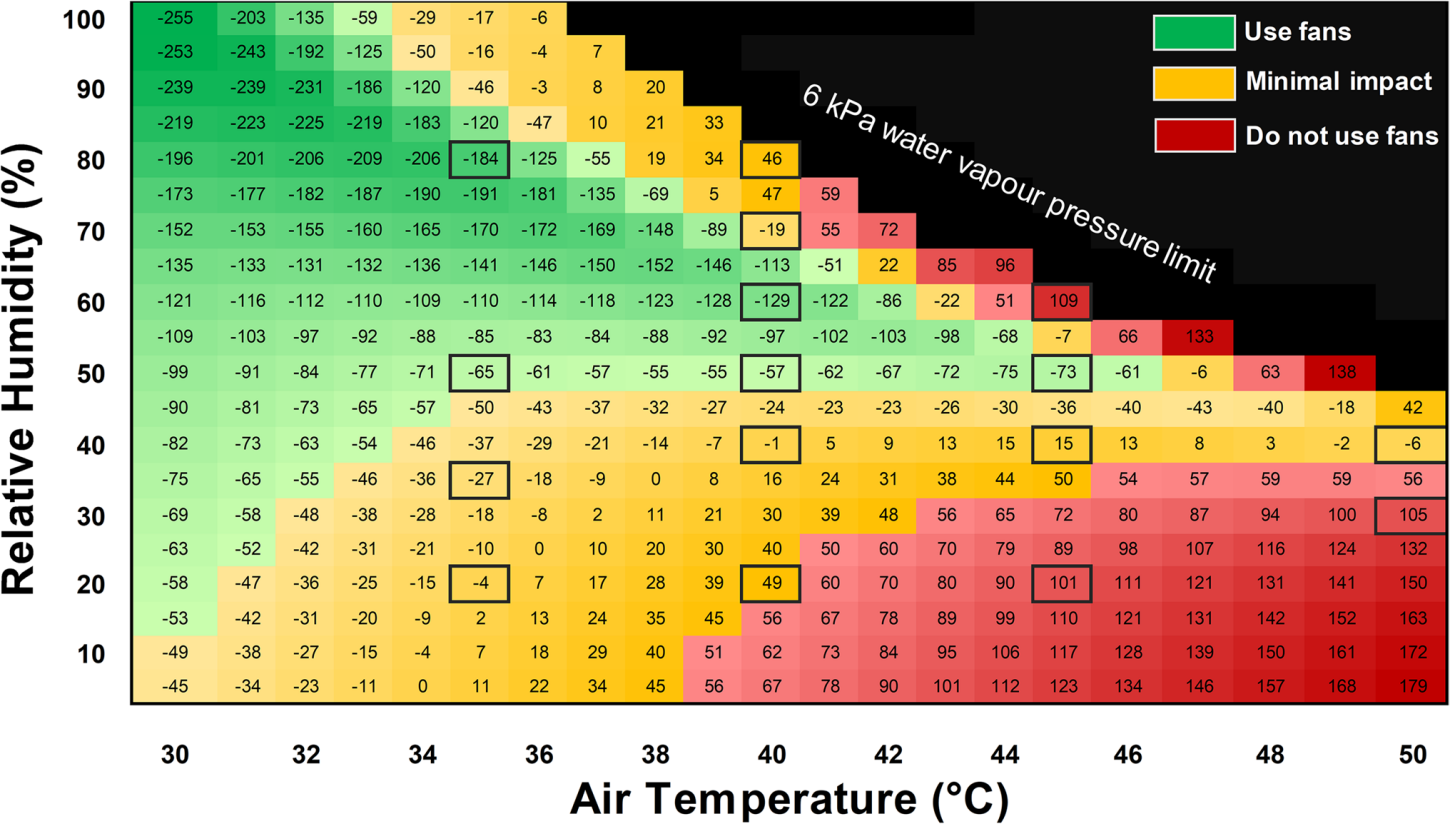
The critical environmental limits for electric fan use during hot work. The numbers in the matrix represent the modelled *change* in body heat storage rate (ΔS, W·m^−2^) elicited by switching on fans, compared with no fan use, i.e. negative numbers = heat loss by switching on fans. The colour zones were determined based on the relationship between the empirical data and the modelled data (see Fig. 3). Based on the linear model in Fig. 3, the green zone indicates where fans are likely to improve work capacity by > 5% (Δ*S* =  < 50 W·m^−2^). The red zones indicate where fans are likely to *decrease* work capacity by > 5% (Δ*S* =  > 50 W·m^−2^). Orange zones indicate where physical work capacity is effectively unchanged by fans (− 50 W·m^−2^ ≤ Δ*S* ≤  + 50 W·m^−2^). The black zone indicates that ambient vapour pressure is ≥ 6 kPa, which has never been reported to arise naturally and is unlikely to be seen even with climate change. The model indicates that fans are detrimental in the black zone, but this is not confirmed with empirical data. The boxes around individual cells indicate the empirical trial conditions used to validate the model (see Fig. 1 for empirical data and Fig. 3 for validation). The assumed sweat rate is equal ~ 1 L per hour, but the equation is shown in the supplementary file Eq. 15. The assumed sweat rate is based on group average data from the empirical trials

The model results are presented for the low clothing coverage condition (0.28 Clo), since it did not account appropriately for clothing factors such as sweat evaporating from the clothing material rather than directly from the skin (Havenith et al., 2013), moisture accumulation in the clothing which can affect clothing heat resistance (Lotens and Havenith, 1995; Wissler and Havenith, 2009), and inconsistencies in wearer fit impacting ventilation through clothing layers (Ke et al., 2013). These sources of error resulted in too much uncertainty in the model output and were ultimately discarded. While the biophysical model is only applicable to conditions involving light clothing, the effect of fans was broadly similar, with the exception that fans were not beneficial at *T*_a_ ≥ 45 ℃ with high clothing coverage. The model for light clothing allowed us to develop a matrix of data which covers all possible combinations of *T*_a_ and relative humidity relevant to current and future occupational heat stress scenarios. The linear regression in Fig. 3 shows the relationship between the ΔPWC with fans (empirical study) and Δ heat storage (model output), for all the temperature and relative humidity conditions tested. In general, the model output agrees well with the empirical data of Fig. 1 (a–d) (*R*^2^ = 0.66), especially when the increased air flow changed heat storage by more than 50 W∙m^−2^. The 50 W∙m^−2^ threshold, based on Fig. 3, is likely to predict a change in PWC of more than 5%. Therefore, this threshold was used to formulate advice within the matrix shown in Fig. 4.

Figure 4 demonstrates the model outcome based on different combinations of *T*_a_ and relative humidity. The green section indicates where fans are beneficial for maintaining PWC. The orange section indicates where fans do not have a strong predicted impact on PWC (± 5% or lower). The red sections indicate where fans are likely to increase heat storage and are therefore not recommended in these zones.

### The efficacy of different heat stress assessment metrics with high wind

**Fig. 5:**
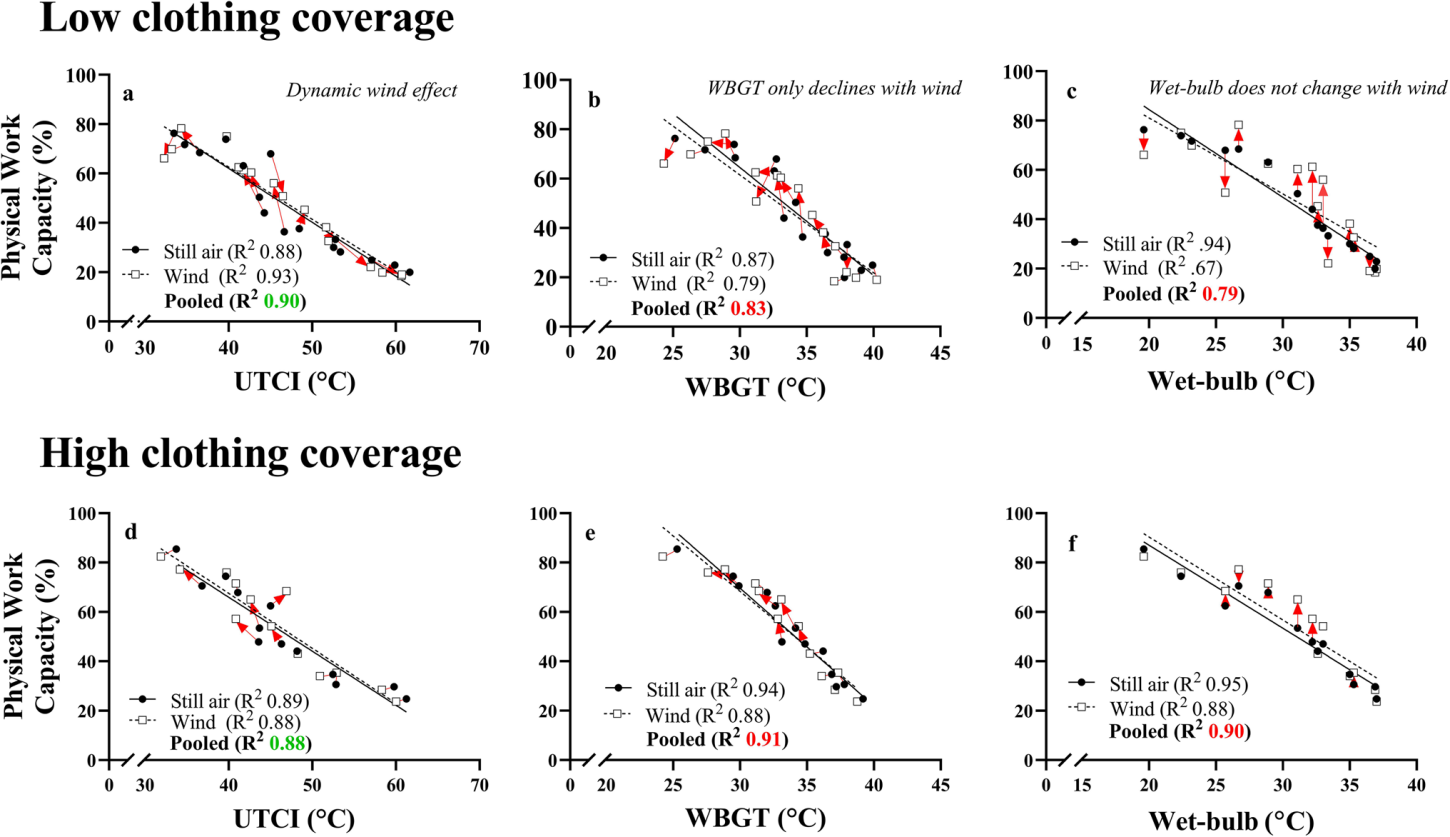
Performance of UTCI, WBGT, and *T*_wb_ when high wind data is included into the model for low (**a**–**c**) and high (**d**–**f**) clothing coverage. Each symbol represents the average observed PWC in a specific *T*_a_/humidity condition, and the arrows point toward the *within subjects* change in PWC with the addition of wind to that condition. In still air conditions, all models showed good predictive power for PWC. In both clothing conditions, the UTCI (**a**) accounts for the dynamic relationship between PWC and humidity in high wind scenarios and showing no loss in predictive power. The WBGT (**b**) and *T*_wb_ (**b**) do not account well for high wind, as shown by the reduced *R*^2^ when high wind data is included into the pooled analysis

Figure 5 plots the full dataset for PWC (*y*) against each heat stress index (*x*). Under still air conditions (no fan), the relationship between PWC and each index suggests that all indices adequately predict PWC (*R*^2^ between 0.88 and 0.93). However, addition of the fan data decreased the predictive capacity of WBGT and *T*_wb_, suggesting that they do not correctly account for added wind. The loss in predictive power is not surprising, since the wind can only ever *decrease* the value of WBGT, and does not change *T*_wb_, while PWC both decreases and increases with wind for different conditions. The UTCI showed no such reduction in predictive power, since it accounts for the dynamic (nonlinear) physiological relationship of wind as shown in Fig. 2**,** with both right and left shifts in UTCI with wind, depending on the conditions, as observed in the empirical data. For example, at *T*_a_ = 40 °C with 60% relative humidity, UTCI *decreased* by 1.5 °C in the presence of wind, reflecting less thermal strain. However, wind *increased* UTCI by similar magnitude at *T*_a_ = 45 °C with 20% relative humidity, reflecting greater thermal strain as supported by our data and others (Morris et al., 2019).

## Discussion

The aim of this study was to document the impact of air movement (wind/electric fans) (3.5 m·s^−1^ vs still air) on physical work capacity in a wide range of air temperature (*T*_a_) and relative humidity combinations. The outcome was assessed with a dual approach of 300 empirical trials in climatic chambers and the development of a biophysical model of human heat transfer. The main outcome was that, at any given *T*_a_ =  ≥ 35 °C, the impact of fans was critically dependent on relative humidity. Fans were typically only beneficial for relative humidity ≥ 50%, suggesting that high wind may be an effective countermeasure against human heat stress in predominantly humid climates. In hot and dry heat, where relative humidity does not meet or exceed 50%, electric fans either had a trivial impact on PWC or were in fact detrimental.

The agreement between the empirical data (Fig. 1) and the biophysical model (Fig. 4) provide compelling evidence that high wind can produce a net cooling effect when *T*_a_ ≥ 35 °C, but the relative humidity must be considered. For the model, the red sections in Fig. 4 can be divided into two main causative domains. The top half is linked to absolute limits in evaporative capacity due to the climate conditions, which at very high vapour pressures limit any positive effects of wind on sweat evaporation. While simultaneous combinations of such high *T*_a_ and relative humidity are rare in natural environments, the emergence of such humid heat is increasing with climate change (Raymond et al., 2020). As such, documenting the impact of air flow in these conditions may have a stronger application for future climate conditions, or in selected uncommon industrial settings. The bottom half, on the other hand, represents an area where negative effects are linked to the physiological sweat production level, where due to the dry climate, all sweat produced will evaporate even in still conditions, and thus wind does not provide a benefit for evaporative heat loss but will still increase convective heat gain (Morris et al., 2019). Based on these findings, and supported by prior work in resting individuals (Morris et al., 2019), fans are not recommended in hot-dry environments, and alternative solutions to mitigate occupational heat stress are recommended. Such solutions include wearing pre-wetted clothing (Cramer et al., 2020), pre-cooling with cold water immersion (Bongers et al., 2015), and pre-cooling with cold fluids or ice slurries (Siegel et al., 2010; Watkins et al., 2018). Ingestion of cold fluids and ice slurry *during* activity can disrupt sweat responses, but is still likely to have a net cooling effect in humid environments (Jay and Morris, 2018).

**Fig. 6:**
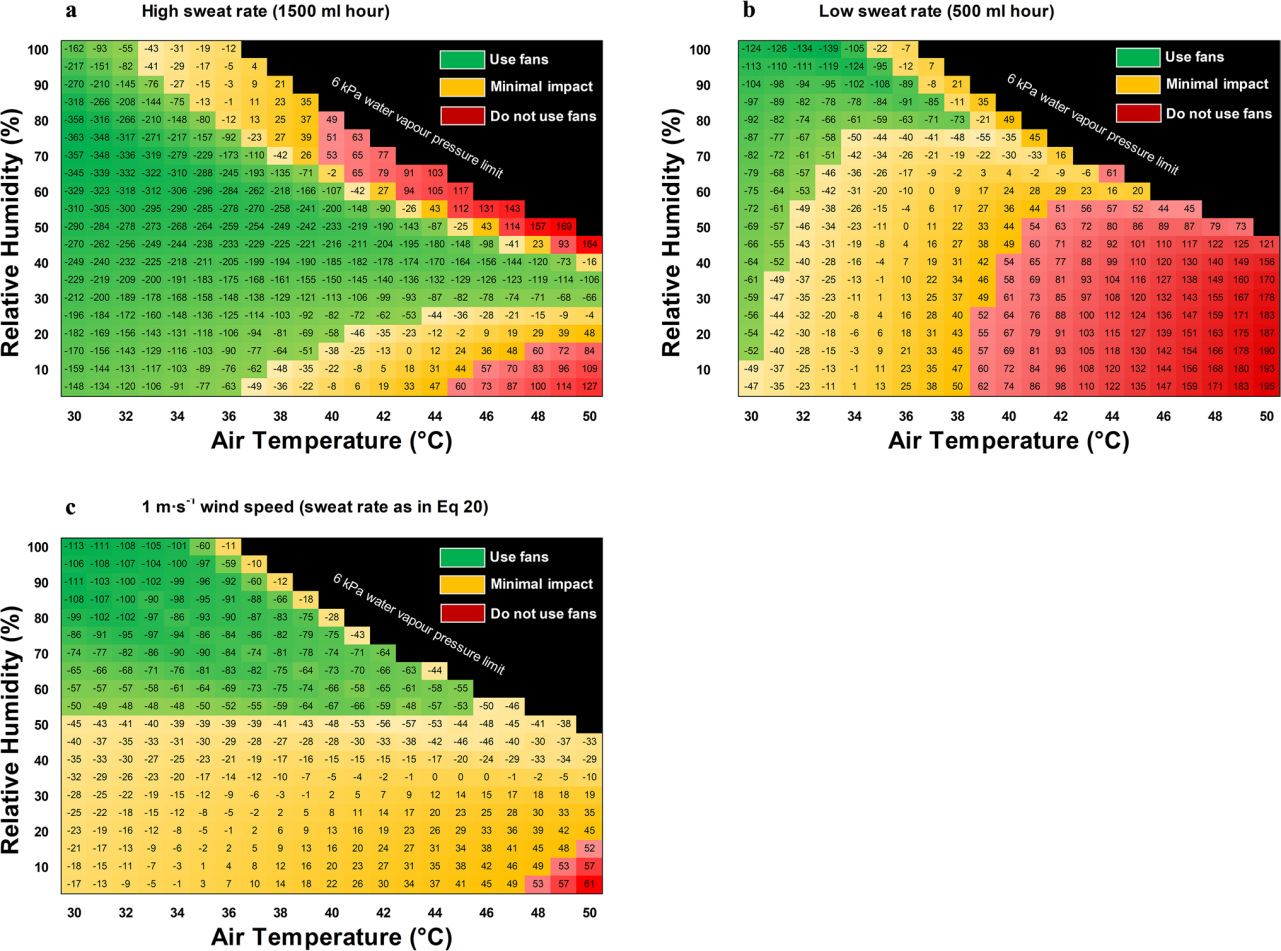
Biophysical models predicting the air temperature and relative humidity combinations in which electric fans are beneficial (green zone), detrimental (red zone), or have a marginal impact on physical work capacity (orange zone). Matrix (**a**) represents a heat acclimatized individual with a sweat rate of 1500 g·h^−1^. Matrix (**b**) represents an individual with a blunted sweat rate of 500 g·h^−1^. Matrix (**c**) used the empirical sweat rate of ~ 1 L per hour as in supplementary file (Eq. 15), with a reduced wind speed of 1 m·s^−1^

The climate zones in which electric fans are beneficial may be altered with different populations. Maximum sweat production is a key factor in determining the impact of fans when *T*_a_ =  ≥ 35 °C since evaporative heat loss provides the only pathway for heat loss. Heat acclimatization and ageing can have a strong influence on sweat production at rest and during physical activity and therefore are likely to alter the results shown in Fig. 4. A period of heat acclimation/acclimatization *increases* sweating sensitivity (faster onset time) and maximum sweat output (Havenith, 2001; Ravanelli et al., 2019; Senay et al., 1976; Smith and Havenith, 2019), whereas ageing can *decrease* total sweat output (Anderson and Kenney, 1987; Coull et al., 2020; Hellon et al., 1956). That the outcome of our model is dependent on sweat rate (Eq. 15 supplementary material) suggests that the latter populations may not experience benefits of fans in the same climatic zones. For example, at rest, during a ramped humidity protocol, there were no benefit of fans on heat strain at *T*_a_ = 42 °C in an elderly population (Gagnon et al., 2016), despite benefits in young people in the same condition (Ravanelli et al., 2015). The impact of sweat rate on the model outcome is shown in Fig. 6.

On average, sweat rates in our young but unacclimatized population were ~ 1000 g·h^−1^ (as used in Fig. 4 matrix), the amount typically expected from young people performing heavy work in the heat (ISO7933, 2004). For illustrative purposes, we show additional model outcomes for elevated sweat rates (1500 g·h^−1^) and depressed sweat rates (500 g·h^−1^), representing heat acclimatized and populations with blunted sweat responses, respectively (Fig. 6). Such blunted sweat response can occur with natural ageing (Inoue et al., 1999), multiple sclerosis (Allen et al., 2017), and spinal cord injury (Griggs et al., 2019). The higher sweat rate in heat acclimatized workers (1500 g·h^−1^) has the largest impact in the hot dry zones, generally reducing the lower relative humidity limit for fans to yield a net cooling effect. Although the potential benefits of wind/fans are also extended to a higher *T*_a_ of 50 °C with heat acclimation, a sweat rate of 1500 g·h^−1^ is extreme for occupational workloads and unlikely to be replaced with water/electrolyte consumption over the course of a full day (Kalkowsky and Kampmann, 2006). Such sweating response may be expected, however, for transient work periods of extreme heat stress (Malchaire et al., 2001). In contrast, a reduced maximum sweat rate to 500 g·h^−1^, which may represent the elderly population, shows that wind/fans have no benefit when *T*_a_ =  ≥ 38 °C. Even at 35 °C, fan benefits are unlikely unless the environment is exceptionally humid (> 80%). The findings from Fig. 6b are consistent with prior empirical research described above (Gagnon et al., 2016). We also show the impact of lower air velocity (1 m·s^−1^ vs still air) (Fig. 6c). Compared with high wind speeds (Fig. 4), low wind speed only has a marginal impact on heat transfer below 50% relative humidity. However, they are unlikely to have a detrimental impact on PWC in most hot working conditions. The simulations shown in Fig. 6 require empirical validation.

Our dataset also allowed us to also document the utility of commonly used environmental heat stress indices (UTCI, WBGT, and *T*_wb_) for the prediction of PWC with forced convection. In still air, PWC decreased linearly with increased relative humidity at each level of *T*_a_ (Fig. 2), reflecting the increase in thermal strain typically associated with increased ambient humidity (Maughan et al., 2012; Sobolewski et al., 2021). Conversely, the best fit curve to the fan data were almost always curvilinear, caused by interaction of sweat rate, sweat evaporation capacity, sweat evaporation efficiency, and humidity. The observation that physiological parameters played a role in the impact of air movement triggered us to test how different heat stress indices account for this curvilinear aspect. Heat stress indices combine different aspects of the environment (typically *T*_a_, humidity, radiation, and wind speed) into one integrated temperature (Havenith and Fiala, 2015) and are typically used in global climate models as an “overall” index of the thermal stress of the environment. For example, the wet-bulb globe temperature (WBGT) and aspirated (psychrometric) wet-bulb temperature (*T*_wb_) (Dunne et al., 2013; Pal and Eltahir, 2016; Raymond et al., 2020) have been incorporated into climate models to assess human adaptation to different climatic scenarios. To accurately link complex human responses to the thermal climate, it is preferable to choose a heat stress index which can account for the dynamic interaction effect of wind with physiological response (Vanos et al., 2020). We demonstrate this by plotting the full dataset against three heat stress indices, namely the WBGT, the *T*_wb_, and the universal thermal climate index (UTCI) (Bröde et al., 2012) (Fig. 5). The output temperature for UTCI is based on a complex thermophysiological model of the human heat stress response and, in theory, should capture both the positive and negative impact of wind on PWC, depending on the climate type, given its inclusion of the physiological components of the heat stress response. Figure 5 demonstrates the utility of each index to account for high wind speed. The red arrows point toward the *change* in PWC elicited by high wind/fans, for the same group of workers (i.e. *within subjects*). The UTCI is the only metric where the PWC prediction improves when including the high wind conditions. Arrows pointing left and right demonstrate that the UTCI value can increase or decrease with high wind, depending on the climate, similar to what we see in the physiological response. In contrast, the WBGT always decreases with wind, while *T*_wb_ does not change. Consequently, the validity of WBGT and *T*_wb_ for predicting PWC in high wind is poor, especially in hot and dry climates.

Any heat stress index which does not account for the dynamic impact of wind due to an interaction with the physiological response should be used with caution when making recommendations about fan use or in climate models that account for wind speed. For example, the US Environmental Protection Agency use the heat index to advise on electric fan use, stating a heat index of 37.2 °C “increases the heat stress the body must respond to” (US Environmental Protection Agency, Excessive Heat Events Guidebook 2006). However, Morris et al. (Morris et al., 2019) demonstrated that for resting individuals, fans were beneficial in a humid climate where the heat index was 55 °C, yet harmful in a hot dry condition where the heat index was 8 °C lower. Similar findings are clear in our data where fans were typically beneficial in humid climates and harmful in hot dry ones. The heat index, like *T*_wb_, does not change with different wind speeds, so cannot be used to inform guidelines relating fan use to thermal climates. Overall, we recommend the UTCI as a preferred heat stress index when assessing the impact of the climate on human physiology.

In summary, the combined impact of the empirical data and the modelled data support three major revisions in public and occupational health guidance related to heat exposure. Firstly, advice for the use of fans cannot be based on *T*_a_ alone since humidity is a critical factor in determining if fans are effective for reducing occupational heat stress. Secondly, the advised threshold of *T*_a_ ≤ 35 °C for fan use is too conservative for young adults during physical work and can be increased to up to *T*_a_ = 45 °C with special caveats relating to relative humidity (see Fig. 4). Finally, we show that electric fans are an ineffective and potentially harmful method for mitigating occupational heat stress in hot-dry conditions. Future work should focus on practical, low cost cooling solutions in these environments.

## Supplementary Information

Below is the link to the electronic supplementary material.

Supplementary file1 (PDF 257 KB)
