# Supplementary material for: Quantifying the impact of heat on human physical work capacity; part II: the observed interaction of air velocity with temperature, humidity, sweat rate, and clothing is not captured by most heat stress indices
Source: Int J Biometeorol. 2021 Nov 6;66(3):507–20. doi: 10.1007/s00484-021-02212-y (PMC8850241; doi:10.1007/s00484-021-02212-y)
Supplement: Supplementary file 1 — Supplementary file1 (PDF 257 KB) [file 484_2021_2212_MOESM1_ESM.pdf]

This supplementary online material includes:

1. A comprehensive description of the heat balance model used in the main text.
2. A table showing the environments used for the empirical study, average trial duration, and number of wind vs no wind comparisons.

Human heat balance modelling

A human biophysical model was used to compare the changes in the rate of heat storage (ΔS) for each air temperature (30 – 50°C) and relative humidity (5 – 100%) combination, with and without fans (i.e. 0.2 vs 3.5 $m \cdot s^{-1}$ air velocity). The model simulation was run based on a worker with low clothing coverage (0.28 Clo). Height and mass were 185cm and 70kg, respectively.

Manually inputted variables

**Variable name**	**Abbreviation**	**Unit**
Air Temperature	T_a	°C
Relative Humidity	*RH*	% (0-100)
Metabolic rate	*M*	$W \cdot m^{-2}$
Sweat rate		$g \cdot hour^{-1}$
Height		cm
Mass		kg
Effective radiative area	A_r	m^{-2}
Intrinsic clothing insulation	*Clo*	*Clo*

The heat balance equation requires input from all physical sources of heat transfer:

$$S = (M - W) - K + C + R + E + C_{res} + E_{res} \text{ [W·m}^{-2}\text{]} \quad \text{Eq 1}$$

All units are expressed in watts per square metre of skin surface area (W·m^{-2}). Where *M* is metabolic energy expenditure; *W* is external work; *K* is conductive heat transfer; *C* is convective heat transfer, *R* is radiative heat transfer; C_{res} and E_{res} are represent convective and evaporative heat transfer from the respiratory tract; *E* is evaporative heat transfer. *W*, *K*, and *R* were assumed to be zero in our analysis. External work to the treadmill is assumed to be negligible, conductive heat transfer from a solid object is negligible, and we assume no sources of radiative heat gain or loss.

<u>Metabolic heat production (*M*)</u>

The main output of the model is the *difference* in heat storage between the fan and no-fan conditions. Therefore, the choice of the *M*-*W* input does not impact the output of the model, as-long as it is set equal between the fan and no-fan condition.

<u>Dry heat transfer calculation</u>

The rate of dry heat (*C* + *R*) transfer was calculated as (Holmér et al., 1999):

$$\frac{(T_{skin} - t_o)}{I_{T,r}} \text{ [W·m}^{-2}\text{]} \quad \text{Eq 2}$$

Where T_{skin} is the mean skin surface temperature; t_o is the operative temperature, and $I_{T,r}$ is resultant thermal insulation. T_{skin}, the average value across a 1-hour trial, was calculated by modelling the empirical data collected in this study:

$$t_{\text{skin}} = 25.883 + (0.23 * T_{\text{a}}) + (0.024 * RH) - (0.304 * Clo)[°\text{C}] \quad \text{Eq 3}$$

Where T_{skin} is mean skin temperature across the body, T_a is ambient temperature in degrees Celsius, and RH is relative humidity (0-100). The predictive capacity of the model was $R^2 = 0.76$. Note this formula was extrapolated for conditions outside of the range of our empirical dataset (see main text Figure 4).

t_o was calculated as (Parsons, 2010):

$$T_o = \frac{(h_r \cdot T_r + h_{c,fan-off} \cdot T_a)}{(h_r + h_{c,fan-off})} \text{ [°C]} \quad \text{Eq 4}$$

Where h_r and $h_{c,fan-off}$ are radiative and convective heat transfer coefficients in fan-off conditions (i.e. still air, not affected by added wind); T_r is mean radiant temperature in degrees Celsius, and was assumed equal to T_a.

h_r was calculated by (Parsons, 2010):

$$h_r = 4\varepsilon\sigma \frac{A_r}{A_D}\left[273.2 + \frac{t_{sk}+t_r}{2}\right]^3 \text{ [W·m}^{-2}\text{·K}^{-1}\text{]} \quad \text{Eq 5}$$

Where ε is the area-weighted emissivity of the clothed body surface, assumed to be 0.95 (ND); σ is the Stefan-Boltzmann constant, equal to 5.67×10^{-8} (W·m^{-2}·K^{-4}); A_r is the effective radiative area of the body in m^{-2}; A_D is the body surface area in m^{-2}; A_r/A_D is typically estimated as 0.77 for a standing person (ISO 7933, 2004).

$h_{c,fan-off}$ was calculated as (Kerslake, 1972):

$$h_{c,fan-off} = 8.3\sqrt{v_{eff,fan-off}} \text{ [W·m}^{-2}\text{·K}^{-1}\text{]} \quad \text{Eq 6}$$

Where $v_{eff,fan-off}$ is the effective air velocity in fan-off wind conditions, calculated by (Lotens and Havenith, 1991):

$$v_{\text{eff,fan-off}} = v_o + v_{wind} + v_{act} \text{ [m·s}^{-1}\text{]} \quad \text{Eq 7}$$

Where v_o is a lower limit related to natural air movement; v_{act} is the rate of motion; v_{wind} is the air motion relative to the body. The following expressions are used (Havenith et al., 1990b):

$$v_o = 0.07 \text{ (sitting) or } 0.11 \text{ (standing)}$$

$$v_{\text{wind}} = \text{measured wind speed}$$

$$v_{\text{act}} = 0.67 v_{\text{walk}} \text{ for treadmill walking or } 0.0043 \times \text{rpm for ergometer cycling}$$

.

The value for v_o was set as 0.11 m·s^{-1}; v_{wind} was set at 0.20 m·s^{-1}and 3.5 m.s^{-1} v_{walk} was set at 1.66 m·s^{-1} (6 km·h^{-1}).

$I_{T,r}$ was calculated as (Havenith et al., 1990b):

$$I_{\text{T,r}} = I_{\text{T,fan-off}} \cdot CorrI_{\text{T}} \text{ [m}^2\text{·K·W}^{-1}\text{]} \quad \text{Eq 8}$$

Where $I_{T,fan\text{-}off}$ is the total insulation in fan-off conditions; $CorrI_T$ is a correction factor (0-1) for $I_{T,fan\text{-}off}$ based on wind speed and walking speed (Havenith and Nilsson, 2004).

$I_{T,fan\text{-}off}$ was calculated as (Parsons, 2010):

$$I_{\text{T,fan-off}} = I_{\text{cl,fan-off}} + \frac{I_{\text{a,fan-off}}}{f_{\text{cl}}} \text{ [m}^2\text{·K·W}^{-1}\text{]} \quad \text{Eq 9}$$

Where $I_{a,fan\text{-}off}$ is the insulation provided by the air layer in fan-off conditions. The correction equation based on wind speed and walking speed is as follows (Havenith and Nilsson, 2004); $I_{cl,fan\text{-}off}$ is the intrinsic thermal resistance of clothing in fan-off conditions (assumed 0.023

$m^2 \cdot K \cdot W^{-1}$); f_{cl} is the clothing area factor (ND), the surface area of the clothed body divided by the nude body surface area.

I_a was calculated as (Parsons, 2010):

$$I_{\text{a,fan-off}} = \frac{1}{h_{static}} \; [m^2 \cdot K \cdot W^{-1}] \qquad \text{Eq 10}$$

With $h_{fan\text{-}off}$ being the combined convective and radiative heat transfer coefficient, while $I_{\text{cl,fan-off}}$ expressed in $m^2 \cdot K \cdot W^{-1}$, f_{cl} is calculated as (Parsons, 2010):

$$f_{\text{cl}} = 1 + 1.81 I_{\text{cl,st}} \; [\text{ND}] \qquad \text{Eq 11}$$

The equation for $CorrI_T$ is dependent on the initial value for $I_{\text{cl,fan-off}}$ (Havenith, 2004). For light clothing ($0.093 < I_{\text{cl,fan-off}} < 0.217$, or $0.6 < I_{\text{cl,fan-off}}$ (clo) < 1.4), $CorrI_T$ was calculated as (Havenith, 2004):

$$CorrI_{\text{T}} = e^{[-0.281(v_{\text{wind}}-0.15)+0.044(v_{\text{wind}}-0.15)^2-0.492v_{\text{walk}}+0.176v_{\text{walk}}^2]} \; [\text{ND}] \qquad \text{Eq 12}$$

Where v_{wind} is the wind speed in $m \cdot s^{-1}$; v_{walk} is the walking speed in $m \cdot s^{-1}$. The value for v_{wind} was set as 0.2 and 3.50 in the still air and fan conditions, respectively. The value for v_{walk} was set as 1.66 $m \cdot s^{-1}$. The equation has only been validated up to 3.5 $m \cdot s^{-1}$ wind speed and 1.2 $m \cdot s^{-1}$ walking speed. For a completely nude body ($I_{cl} = 0$), CorrI_T becomes a correction for air insulation only, CorrI_a (Havenith and Nilsson, 2004):

$$CorrI_{\text{T}} = CorrI_{\text{a}} = e^{[-0.533(v_{\text{wind}}-0.15)+0.069(v_{\text{wind}}-0.15)^2-0.462v_{\text{walk}}+0.201v_{\text{walk}}^2]} \; [\text{ND}] \qquad \text{Eq 13}$$

$I_{\mathrm{T,r}}$ is then calculated as in Eq 8. For very light clothing ($0 < I_{\mathrm{cl,st}} < 0.093$ or $0 < I_{\mathrm{cl,st}}$ (clo) $<$ 0.6) an equation for interpolation between Eq's 12 and 13 was developed (Havenith et al., 2000). All units below are in $\mathrm{m^2{\cdot}K{\cdot}W^{-1}}$.

$$I_{\mathrm{T,r}} = \frac{[(0.093 - I_{\mathrm{cl}})I_{\mathrm{T,r\ nude}} + (I_{\mathrm{cl}} \cdot I_{\mathrm{T,r\ clothed}})]}{0.093}\ [\mathrm{m^2{\cdot}K{\cdot}W^{-1}}] \qquad \text{Eq 14}$$

Correction equations specifically for low wind speeds ($0.4 – 1\ \mathrm{m{\cdot}s^{-1}}$) or for heavy protective clothing are available (Havenith, 2004).

With $I_{\mathrm{T,r}}$ solved, the rate of dry heat transfer can be calculated as in Eq 2.

Evaporative heat transfer calculation

The rate of evaporative heat loss from sweat (E_{sweat}) was calculated based on actual sweat production from the empirical trials, which was very similar across conditions, but varied slightly as a function of water vapour pressure. The sweat rate was inputted into the model to capture the pattern shown in the empirical data:

$$Sweat\ rate = (459.5 - 1000)e^{(-0.519 \cdot P_{\mathrm{a}})} + 1000\ [\mathrm{g{\cdot}hour^{-1}}] \qquad \text{Eq 15}$$

Where Pa is ambient water vapour pressure, measured by:

$$P_a = e^{\left(18.956 - \frac{4030.18}{T_a + 235}\right)} \times \frac{RH}{100}\ \ [\mathrm{kPa}] \qquad \text{Eq 16}$$

Due to the reduction in work rates in hotter climates, most conditions elicited a sweat rate of ~1000 $\mathrm{g{\cdot}hour^{-1}}$. The average sweat rate across all conditions was 977 ± 236 $\mathrm{g{\cdot}hour^{-1}}$ and is the maximum that can be expected from an average worker undertaking moderate to heavy work in the heat (ISO7933, 2007). However, in very dry conditions, we found reduced sweat

rates despite higher work rates, prompting us to choose a dynamic model as shown above. The chosen sweat rate is representative of both the empirical data and the maximum of what could be expected in the field in young adults free of thermoregulatory impairment.

E_{sweat} was calculated as (Malchaire et al., 2001):

$$E_{sweat} = Sw_{\max} \cdot \eta \ [\mathrm{W \cdot m^{-2}}] \qquad \text{Eq 17}$$

Where $Sw_{\max}$ is theoretical maximum possible *evaporative* heat loss from sweat, assuming all sweat produced evaporates into the environment; η is sweating efficiency i.e. the actual proportion of sweat that evaporates to provide a heat loss effect (0-1) i.e. if $\eta = 0.5$, 50% of sweat evaporates (providing evaporative cooling) and 50% drips (providing a negligible heat loss effect).

$Sw_{\max}$ was calculated as:

$$Sw_{\max} = \frac{Sw_{\mathrm{prod}} \cdot Sw_{\mathrm{latent}}}{3600} \ [\mathrm{W \cdot m^{-2}}] \qquad \text{Eq 18}$$

Where Sw_{prod} is sweat produced in $\mathrm{g \cdot m^{-2} \cdot hour^{-1}}$, where A_D was set as 1.92 $\mathrm{m^{-2}}$, representing the average of all participants in our study; Sw_{latent} is the latent heat of vaporisation of sweat, set as 2430 $\mathrm{J \cdot g^{-1}}$ (Wenger, 1972). This represents the heat energy lost from the body per gram of sweat that evaporates into the environment. 3600 is an adjustment factor for time i.e., one hour to one second.

η was calculated as (Malchaire et al., 2001):

$$\eta = 1 - \frac{w^2}{2} \quad \text{for } w \leq 1$$

$$\eta = \frac{(2-w)^2}{2} \quad \text{for } 1 < w \leq 1.7 \text{ [ND]} \qquad \text{Eq 19}$$

$$\eta = 0.05 \text{ for } w > 1.7$$

Where w is the skin wettedness i.e., proportion of A_D covered with sweat. Values of w that exceed 1 can be interpreted as a growing thickness of the sweat layer on the skin (Malchaire et al., 2001).

w is calculated as (Parsons, 2010):

$$w = \frac{Sw_{\text{max}}}{E_{\text{max}}} \text{ [ND]} \qquad \text{Eq 20}$$

Where E_{max} is the theoretical maximum evaporative heat loss possible, dictated by the environmental humidity and clothing evaporative resistance. It is calculated by (Havenith et al., 1999):

$$E_{\text{max}} = \frac{P_{\text{sk,s}} - P_{\text{a}}}{R_{\text{e,T,r}}} \text{ [W·m}^{-2}\text{]} \qquad \text{Eq 21}$$

Where $P_{sk,s}$ is the saturated water vapour pressure at skin temperature in kPa; P_a is the ambient water vapour pressure at air temperature in kPa; $R_{e,T,r}$ is the resultant total evaporative resistance of clothing. $P_{sk,s}$ was calculated by (Parsons, 2010):

$$P_{\text{sk,s}} = \frac{e^{\left(18.956 - \frac{4030.18}{t_{\text{skin}} + 235}\right)}}{10} \text{ [kPa]} \qquad \text{Eq 22}$$

P_a was calculated as in Eq 16. $R_{e,T,r}$ was calculated by (Havenith et al., 1990b):

$$R_{e,T,r} = R_{e,T,fan-off} \cdot CorrR_{e,T} \text{ [m}^{-2}\cdot\text{kPa }\cdot\text{W}^{-1}\text{]} \quad \text{Eq 23}$$

Where $R_{e,T,fan-off}$ is the total evaporative resistance of clothing in fan-off conditions. With clothing > 0.6 Clo, $CorrR_{e,T}$ is a correction factor (0-1) for $R_{e,T,fan-off}$ based on wind speed and walking speed (Havenith et al., 1990a). $R_{e,T,fan-off}$ is calculated as (ISO9920, 2009):

$$R_{e,T,fan-off} = R_{e,cl,fan-off} + \frac{R_{e,a,fan-off}}{f_{cl}} \text{ [m}^{-2}\cdot\text{kPa}^{-1}\cdot\text{W}^{-1}\text{]} \quad \text{Eq 24}$$

Where $R_{e,cl,fan-off}$ is the intrinsic evaporative resistance of clothing; $R_{e,a,fan-off}$ is the evaporative resistance of the air layer. f_{cl} is calculated as in Eq 11. $R_{,e,cl,fan-off}$ was calculated as (ISO9920, 2009):

$$R_{e,cl,fan-off} = I_{cl,fan-off} \times 0.18 = 0.006 \text{ [m}^{-2}\cdot\text{kPa}^{-1}\cdot\text{W}^{-1}\text{]} \quad \text{Eq 25}$$

$R_{e,a,fan-off}$ was calculated (ISO9920, 2009):

$$R_{e,a,fan-off} = \frac{1}{h_{e,fan-off}} \text{ [m}^{-2}\cdot\text{kPa}^{-1}\cdot\text{W}^{-1}\text{]} \quad \text{Eq 26}$$

Where $h_{e,fan-off}$ is the evaporative heat transfer coefficient in fan-off conditions, calculated as (Parsons, 2010):

$$h_{e,fan-off} = 16.5 h_{c,fan-off} \text{ [W}^{-1}\cdot\text{m}^{-2}\cdot\text{kPa}^{-1}\text{]} \quad \text{Eq 27}$$

For light clothing (see Eq 13 for definition), $CorrR_{e,T}$ was calculated as (ISO9920, 2009):

$$CorrR_{\mathrm{e,T}} = e^{[-0.468(v_{\mathrm{wind}}-0.15)+0.080(v_{\mathrm{wind}}-0.15)^2-0.87v_{\mathrm{walk}}+0.358v_{\mathrm{walk}}^2]}\ [\mathrm{ND}] \quad \text{Eq 28}$$

In completely nude conditions (0 Clo), $CorrR_{\mathrm{e,T}}$ can be disregarded, since $R_{\mathrm{e,T,r}}$ is calculated only based on fan-on $R_{\mathrm{e,a}}$ ($R_{\mathrm{e,a,fan\text{-}on}}$, see Eq 29). For very light clothing, an interpolation is made available in Eq 32. $R_{\mathrm{e,a,fan\text{-}on}}$ can be calculated as:

$$R_{e,T,r} = R_{\mathrm{e,a,fan-on}} = \frac{1}{h_{e,dyn}}\ [\mathrm{m^{-2}\cdot kPa^{-1}\cdot W^{-1}}] \quad \text{Eq 29}$$

Where $h_{\mathrm{e,fan\text{-}on}}$ is the evaporative heat transfer coefficient based on the effective air velocity (Parsons, 2010)

$$h_{e,dyn} = 16.5h_{c,dyn}\ [\mathrm{W^{-1}\cdot m^{-2}\cdot kPa^{-1}}] \quad \text{Eq 30}$$

Where $h_{\mathrm{c,fan\text{-}on}}$ is the convective heat transfer coefficient (Parsons, 2010):

$$h_{\mathrm{c,fan-on}} = 8.3\sqrt{v_{\mathrm{eff,fan-on}}}\ [\mathrm{W\cdot m^{-2}\cdot K^{-1}}] \quad \text{Eq 31}$$

Where v_{eff} is the effective air velocity, calculated as in Eq 7 (Lotens and Havenith, 1991). The value for v_{o} was set as 0.11; v_{wind} was set at 0.2 and 3.50 m·s^{-1}; v_{walk} was set at 1.66 m·s^{-1} (6 km·h^{-1}).

$R_{\mathrm{e,T,r}}$ is then calculated as in Eq 23. But for very light clothing (see Eq 13 for definition) an equation for interpolation between results using equations 28 and 29 is possible. All units below are in m^{-2}·kPa^{-1}·W^{-1}:

$$R_{\mathrm{e,T,r}} = \frac{[(0.093-I_{cl})R_{e,a,dyn}]+(I_{cl}\cdot R_{\mathrm{e,T,r,clothed}})}{0.093}\ [\mathrm{m^{-2}\cdot kPa^{-1}\cdot W^{-1}}] \quad \text{Eq 32}$$

Depending on the $I_{cl,fan\text{-}off}$ value, $R_{e,T,r}$ is solved based on Eq 23, 30, or 32. Solving $R_{e,T,r}$ now allows for calculation of E_{sweat} (Eq 17). The actual value used for E in Eq 1 was lowest value of E_{max} or E_{sweat}.

Respiratory heat transfer calculation

The final parameter in the model is calculation of respiratory heat transfer. Total respiratory heat transfer is the combined effect of dry respiratory heat loss (C_{res}) and latent respiratory heat loss (E_{res}).

C_{res} is calculated from (Parsons, 2010):

$$C_{\text{res}} = 0.0014M \cdot (34 - T_a) \text{ [W·m}^{-2}\text{]} \qquad \text{Eq 33}$$

E_{res} is calculated from (Parsons, 2010):

$$E_{\text{res}} = 0.0173M \cdot (5.87 - P_a) \text{ [W·m}^{-2}\text{]} \qquad \text{Eq 34}$$

Where P_a is the partial pressure of water vapour in air, calculated as (Parsons, 2010):

$$P_{\text{a}} = e^{\left(18.956 - \frac{4030.18}{Ta+235}\right)} \times \frac{RH}{100} \text{ [kPa]} \qquad \text{Eq 35}$$

Determining the effect of fans:

The above equations allow calculation of the rate of heat storage (S) between fan on (3.5 $m \cdot s^{-1}$) and fan off (0.2 $m \cdot s^{-1}$) conditions, for the same $I_{T,fan\text{-}off}$. For fan off and fan on conditions, S was compared for all relevant combinations of air temperature (30-50°C in 1°C steps) and relative humidity (5-100% in 5% steps, up to 6kPa). This resulted in 328 total comparisons of S (i.e., 1 for each combination of air temperature and relative humidity). The difference in S was calculated as:

$$\text{Fan effect } (\Delta S) = S_{fan-on} - S_{fan-off} \text{ [W·m}^{-2}\text{]} \quad \text{Eq 36}$$

Where ΔS is the change in heat storage elicited by increasing wind speed (switching fans on). $S_{\text{fan-off}}$ is the rate of heat storage in still air; $S_{\text{fan-on}}$ is the rate of heat storage in the with fans switched on.

Table S1. Environments used in the empirical study, average trial duration for each condition, and number of *within participant* comparisons completed per environment.

T_a	RH	P_a	Trial duration				Still air vs fan comparisons	
			Low clothing coverage		High clothing coverage		Low clothing coverage	High clothing coverage
			Still air	Fan	Still air	Fan		
15	50	0.8		na			0	0
35	20	1.2					5	4
35	35	1.9			na	na	4	0
35	50	3.1					5	5
35	80	4.7	43		48		5	4
40	20	1.4					6	5
40	40	3.0					7	5
40	50	3.7	47		51		5	5
40	60	4.4	34		44	47	6	5
40	70	5.2	29	40	34	36	5	5
40	80	6.0	20	22	27	25	4	4
45	20	1.9		53	52		4	5
45	40	3.8	37	47	39	43	6	6
45	50	4.8	29	33	31	32	6	5
45	60	5.6	23	24	26	26	3	5
50	30	3.7	36	25	na	na	3	0
50	40	4.8	22	19	na	na	2	0

T_a, air temperature in °C; RH, relative humidity (%); P_a, air vapour pressure in kPa; for trial duration columns, filled boxes indicate that the full trial was completed for all participants.

References

Havenith, G. (2004). “METHOD: THERMAL CONDITIONS MEASUREMENT,” in *Handbook of Human Factors and Ergonomics Methods*, eds. N. Stanton, A. Hedge, K. Brookhuis, E. Salas, and H. Hendrick (CRC Press LLC). doi:10.1201/9780203489925.

Havenith, G., Heus, R., and Lotens, W. A. (1990a). Clothing ventilation, vapour resistance and permeability index: Changes due to posture, movement and wind. *Ergonomics*. doi:10.1080/00140139008925308.

Havenith, G., Heus, R., and Lotens, W. A. (1990b). Resultant clothing insulation: A function of body movement, posture, wind, clothing fit and ensemble thickness. *Ergonomics* 33, 67–84. doi:10.1080/00140139008927094.

Havenith, G., Holmér, I., den Hartog, E. A., and Parsons, K. C. (1999). Clothing evaporative heat resistance—proposal for improved representation in standards and models. *Ann. Occup. Hyg.* 43, 339–346. doi:10.1093/annhyg/43.5.339.

Havenith, G., Holmér, I., Parsons, K., Den Hartog, E., and Malchaire, J. (2000). Calculation of Dynamic Heat and Vapour Resistance. in *9th International Conference on Environmental Ergonomics* (Dortmund, Germany), 125–128.

Havenith, G., and Nilsson, H. O. (2004). Correction of clothing insulation for movement and wind effects, a meta-analysis. *Eur. J. Appl. Physiol.* doi:10.1007/s00421-004-1113-6.

Holmér, I., Nilsson, H., Havenith, G., and Parsons, K. (1999). Clothing convective heat exchange—proposal for improved prediction in standards and models. *Ann. Occup. Hyg.* 43, 329–337. doi:10.1093/annhyg/43.5.329.

ISO9920 (2009). International Standardisation Organisation. Ergonomics of the thermal

environment - Estimation of thermal insulation and water vapour resistance of a clothing ensemble.

Kerslake, D. M. (1972). The stress of hot environments. *Monogr. Physiol. Soc.* doi:10.1016/0013-9351(73)90034-0.

Lotens, W. A., and Havenith, G. (1991). Calculation of clothing insulation and vapour resistance. *Ergonomics* 34, 233–254. doi:10.1080/00140139108967309.

Malchaire, J., Piette, A., Kampmann, B., Mehnert, P., Gebhardt, H., Havenith, G., et al. (2001). Development and validation of the predicted heat strain model. *Ann. Occup. Hyg.* 45, 123–135. doi:10.1016/S0003-4878(00)00030-2.

Parsons, K. C. (2010). *Human thermal environments*. 2nd ed. London: Taylor & Francis doi:10.4324/9780203302620_chapter_1.

Wenger, C. B. (1972). Heat of evaporation of sweat: thermodynamic considerations. *J. Appl. Physiol.* 32, 456–459. doi:10.1152/jappl.1972.32.4.456.
